# Circulating Growth Differentiation Factor 15 (GDF15) in Paediatric Disease: A Systematic Review

**DOI:** 10.1002/jcsm.13712

**Published:** 2025-02-28

**Authors:** David W. Kronenberger, Teresa A. Zimmers, Rick K. Ralston, Daniel V. Runco

**Affiliations:** ^1^ Indiana University School of Medicine Indianapolis Indiana USA; ^2^ Department of Cell, Developmental, and Cancer Biology, Knight Cancer Institute Oregon Health & Science University Portland Oregon USA; ^3^ Ruth Lilly Medical Library Indiana University School of Medicine Indianapolis Indiana USA; ^4^ Division of Hematology/Oncology, Department of Pediatrics, Seattle Children's Hospital University of Washington Seattle Washington USA; ^5^ Ben Towne Center for Childhood Cancer Research Seattle Children's Research Institute Seattle Washington USA; ^6^ Fred Hutchinson Cancer Research Center Seattle Washington USA

**Keywords:** GDF15, growth differentiation factor 15, inflammation, metabolism, paediatric cachexia

## Abstract

**Background:**

Growth Differentiation Factor 15 (GDF15), a nonspecific inflammatory marker and member of the TGF‐β superfamily, has a well‐established role in both inflammation and metabolic modulation, but lacks a comprehensive paediatric literature review. In several adult disease states, including cancer cachexia and pregnancy, circulation and expression of GDF15 has been of clinical and scientific interest, but little published paediatric data exists. As such, we aim to summarize existing paediatric studies.

**Methods:**

This review follows the PRISMA‐ScR guidelines for reporting and aims to summarize existing paediatric studies including GDF15, describe disease entities in which GDF15 has been investigated including existing reference ranges, and identify literature gaps to present future clinical and research direction. Our search strategy queried Ovid MEDLINE, Ovid Embase, Cochrane Library and Scopus databases to find original scientific articles measuring GDF15 from birth through children up to age 18. Data relating to study participant demographic and disease pathology, GDF15 measurement methods and clinical outcomes of interest were extracted.

**Results:**

Sixty‐two studies were included, classified as cardiac, endocrine, mitochondrial, hematologic, neonatal, oncologic, infectious, rheumatologic, renal, neurologic or healthy. While several entities demonstrated elevated GDF15, the highest median GDF15 levels were observed in cardiac arrest 7089 pg/mL (interquartile range 3805–13 306) and mitochondrial diseases 4640 pg/mL (1896–14 064). In certain conditions, including cardiac stress, polycystic ovarian syndrome (PCOS), Kawasaki Disease (KD) and certain mitochondrial myopathies GDF15 can normalize with disease treatment or resolution. Of healthy children studied, GDF15 levels were highest in healthy neonates and followed a predictable pattern, decreasing over time. Mean and standard deviation values of GDF15 in healthy children were 343.8 ± 221.0 pg/mL, with a range of 90–1134 pg/mL for study averages.

**Conclusions:**

Circulating GDF15 has been studied in a variety of paediatric diseases. However, variable evaluated outcome measures and GDF15 measurement methodologies prevent generalizability and direct comparison of these published studies. Validating normal GDF15 levels in children with standardized and reproducible methodology will help clarify GDF15's utility as a diagnostic marker of disease, a necessary step to elucidate clinical implications of GDF15 over expression and its potential as a therapeutic target.

## 1 Introduction

Since being described in 1997, interest in Growth Differentiation Factor 15 (GDF15), a member of the TGF‐β superfamily, has grown due to its multifactorial and occasionally paradoxical roles in metabolism and inflammation [1, 2, 3, 4]. Due its distinct and variable roles, GDF15 has been known by multiple aliases: prostate‐derived factor/prostate differentiation factor (PDF), macrophage inhibitory cytokine‐1 (MIC1), placental bone morphogenetic protein (PLAB), non‐steroidal anti‐inflammatory drug‐inducible gene (NAG‐1) and placental transforming growth factor‐beta (PTGFB) [5]. The only known receptor for GDF15 is glial cell line‐derived neurotrophic factor (GDNF) family receptor α‐like (GFRAL), found exclusively in the hindbrain and the brainstem [4, 6]. Through downstream pathways such as RET, PI3K, MAPK and SMAD2/3, GDF15 signalling exerts a widespread response to stressors [6]. Under physiologic conditions, GDF15 is expressed primarily in placenta, prostate and urothelium, as well as in some cells of the GI tract [4, 7, 8]. GDF15 is important in energy homeostasis, demonstrated by GDF15 knockout mice preferring high‐fat foods and developing insulin resistance compared to healthy mice [6]. Additionally, GDF15 regulates macrophage function, demonstrating importance in regulating appropriate immune and inflammatory response to infection and sepsis [2, 5]. The independent, causal effects of GDF15 on cachexia, inflammation and other diseases remains debated. Further, GDF15 is modulated by various drugs including chemotherapeutics, metformin and non‐steroidal anti‐inflammatory drugs [4, 7, 9]. In such contexts, GDF15 affects maladaptive responses, including anorexia, nausea and vomiting in widely disparate settings: chemotherapy, oncologic immunosuppression, organ failure and hyperemesis gravidarum [10].

Study of GDF15's impact on energy balance and inflammation has led to clinical development of GDF15‐mimetics for obesity and metabolic syndrome and GDF15‐inhibitors or antagonists for cancer‐related cachexia, immunosuppression and heart disease [11]. Despite the growing body of evidence that cachexia worsens morbidity and mortality in adults, no paediatric studies have assessed changes in GDF15 over time as predictors of morbidity and mortality [6]. Novel research in the creation of GFRAL antagonists, thus inhibiting GFRAL dependent GDF15 response, have shown promise in reducing cachexia in in vivo models [12, 13]. Yet, many of the tissues affected by GDF15 do not express GFRAL. Thus, the potential for undiscovered receptors for GDF15 in the body exist. GDF15's function in various pathologies could be GFRAL dependent, independent, or a multi‐receptor mediated. Advancing GDF15 therapeutics to children requires better functional understanding, and knowledge of both normal and pathological levels. However, to our knowledge, no summative description of paediatric studies involving GDF15 exists. Further, recent research has proven variability in measurements of a coding variant of GDF15, yielding up to 60% underreporting of the minor allele variant in some immunoassays including R&D Systems, emphasizing the poor consensus on clinical or research methods of detection available, and even less information about GDF15 variability by age, biologic sex and pubertal status in healthy children or those with disease [14].

With a growing body of literature implicating GDF15 as a driver of energy homeostasis, potentially mediating both obesity and cachexia, elucidating its role in children is imperative to both understanding and changing the childhood obesity epidemic, as well as mediating systemic toxicities of disease. GDF15 might serve adaptive, anti‐inflammatory and metabolically protective functions under normal physiological conditions; however, various pathologies, particularly chronic diseases, demonstrate high circulating levels of GDF15 with worse disease outcomes, which have not fully been explored in the paediatric population [6]. This review aims to provide a comprehensive accounting of the current paediatric studies involving circulating GDF15 and suggest opportunities to understand GDF15's role as a diagnostic marker, mediator and/or therapeutic target in multiple paediatric diseases.

## 2 Methods

**FIGURE 1:**
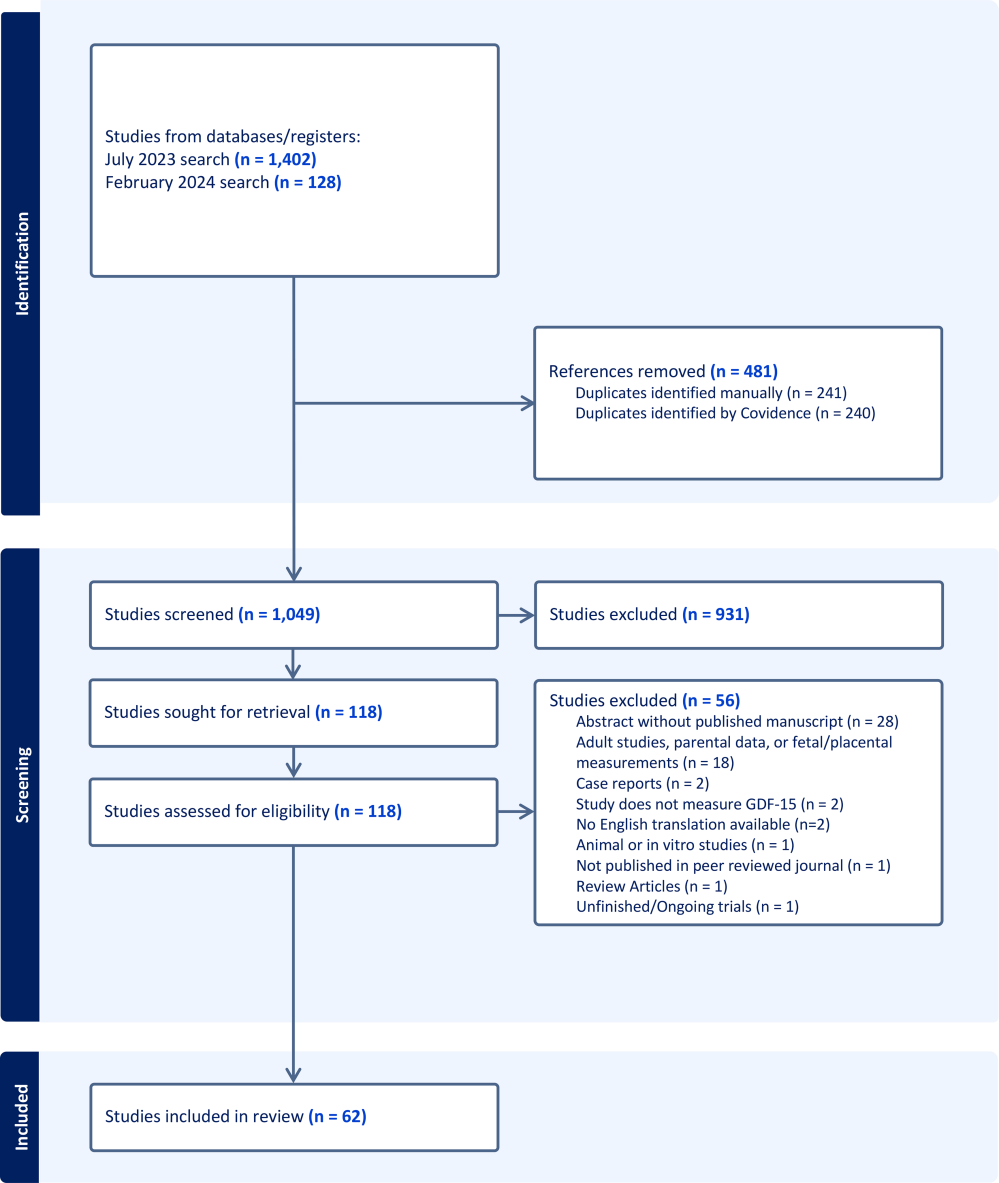
PRISMA‐style flow diagram of search criteria.

A research question was developed and refined utilizing PICO criteria and subsequently we conducted a systematic search in Ovid MEDLINE, Ovid Embase, Cochrane Library and Scopus from inception through 12 February 2024 (Supporting Information Appendix S1). Growth Differentiation Factor 15 and associated terms were used: “GDF15”, “GDF 15”, “growth and differentiation factor 15”, “growth differentiation factor 15”, “prostate‐derived factor”, “prostate differentiation factor”, “MIC1”, “MIC‐1”, “macrophage inhibitory cytokine”, “PLAB”, “placental bone morphogenetic protein”, “NAG‐1”, “non‐steroidal anti‐inflammatory drug‐inducible gene”, “PTGFB”, “placental transforming growth factor‐beta” and “placental TGF beta”. To limit results to GDF15 levels in circulating blood of human children, the following terminology was used: “Adolescen* or Teen* or Youth* or Child* or Infant* or Infanc* or Newborn* or Neonat* or P?ediatric* or PICU* or (Kid or kids) or Toddler*” or “Adolescent/ or exp Child/ or Infant/ or Infant, Newborn/ or Pediatrics/ or Hospitals, Pediatric/or Intensive Care Units, Pediatric/”, and “not (Animals/not (Animals/and Humans/))”. Articles were then screened by reviewers to include only circulating GDF15 assays with reported levels. Titles and abstracts were reviewed for relevance and inclusion criteria by two reviewers, with discrepancies adjudicated between the two. Subsequently identified full text articles were uploaded into Covidence for review and duplicate removal. Inclusion and exclusion criteria were applied to fully reviewed manuscripts (Figure 1).

**FIGURE 2:**
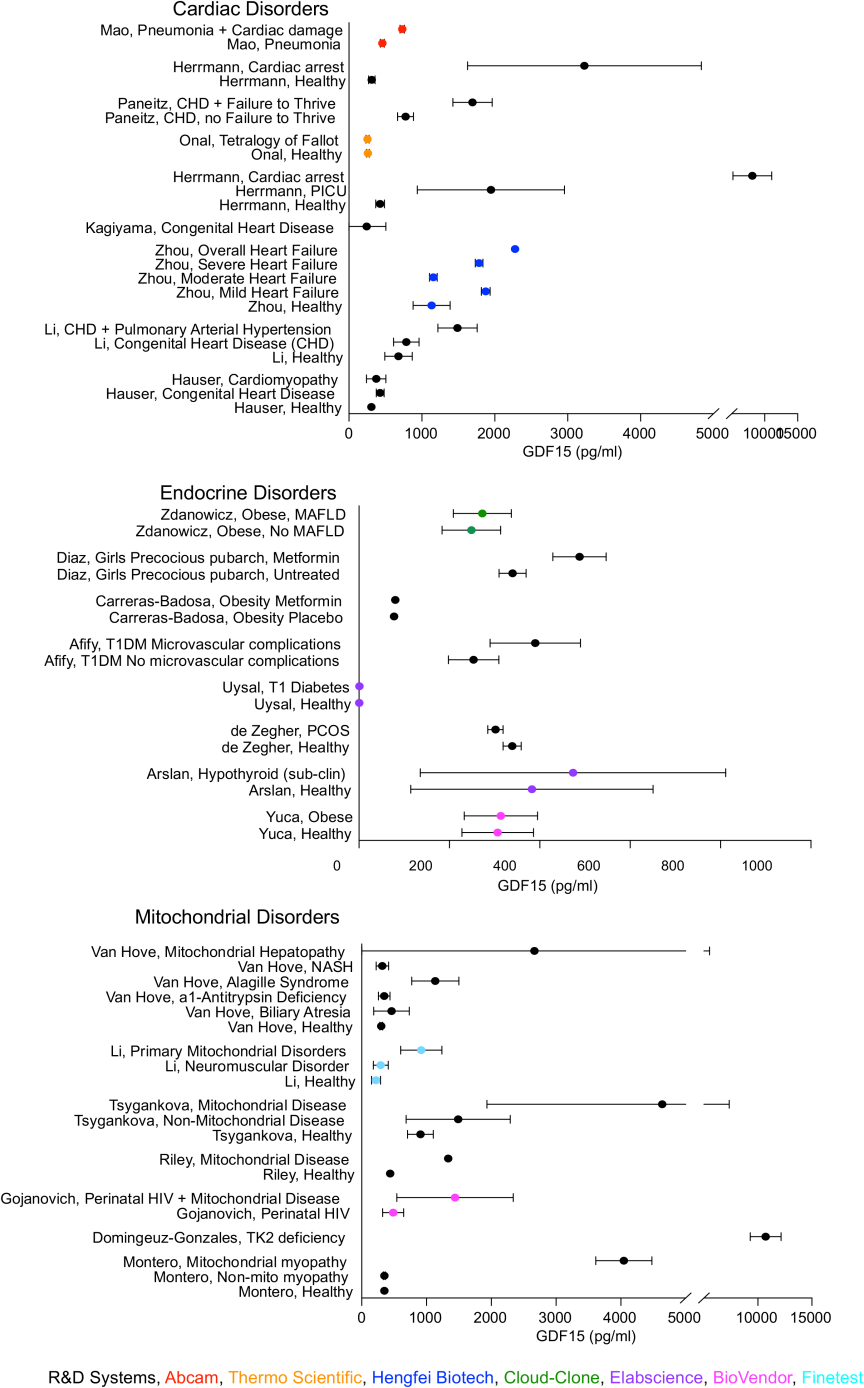
Reported GDF15 levels in cardiac, endocrine, and mitochondrial disorders. Data are reported as mean ± SD. When the references reported median and interquartile range (IQR), mean and SD were calculated from non‐skewed data or approximated as Median ± IQR.

**FIGURE 3:**
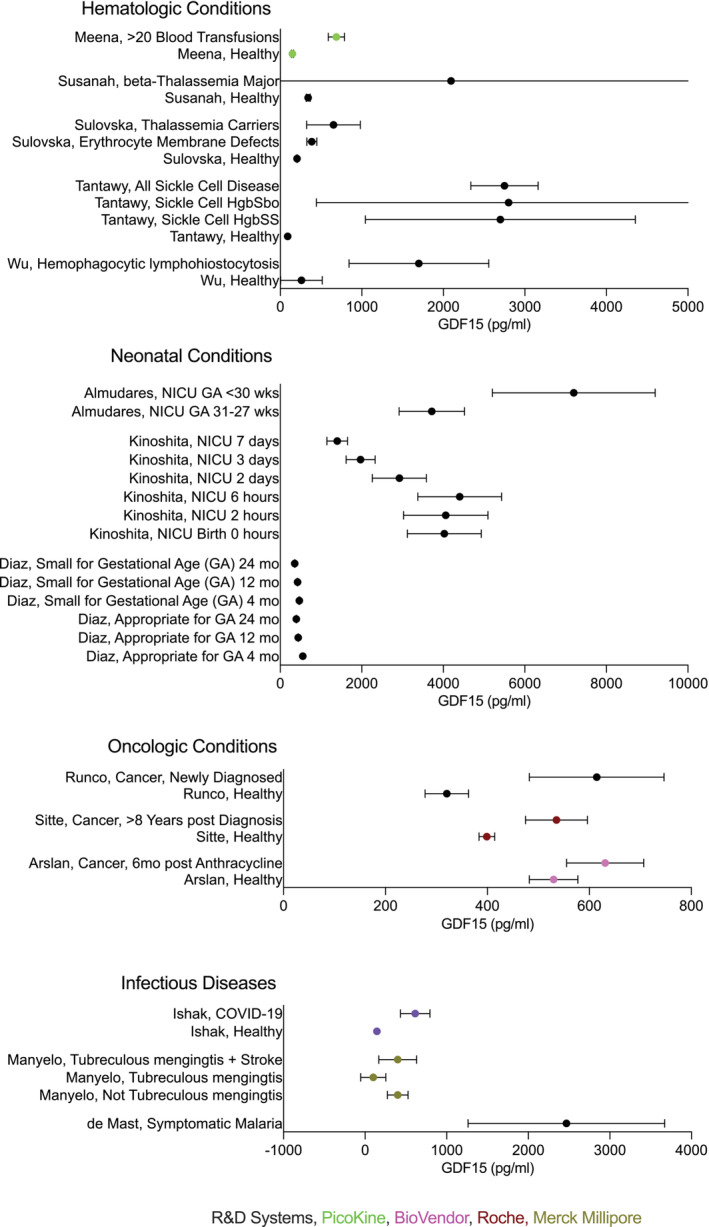
Reported GDF15 levels in hematologic, neonatal, oncologic conditions, and infectious diseases. Data are reported as mean ±SD. When the references reported median and interquartile range (IQR), mean and SD were calculated from non‐skewed data or approximated as Median ± IQR.

**FIGURE 4:**
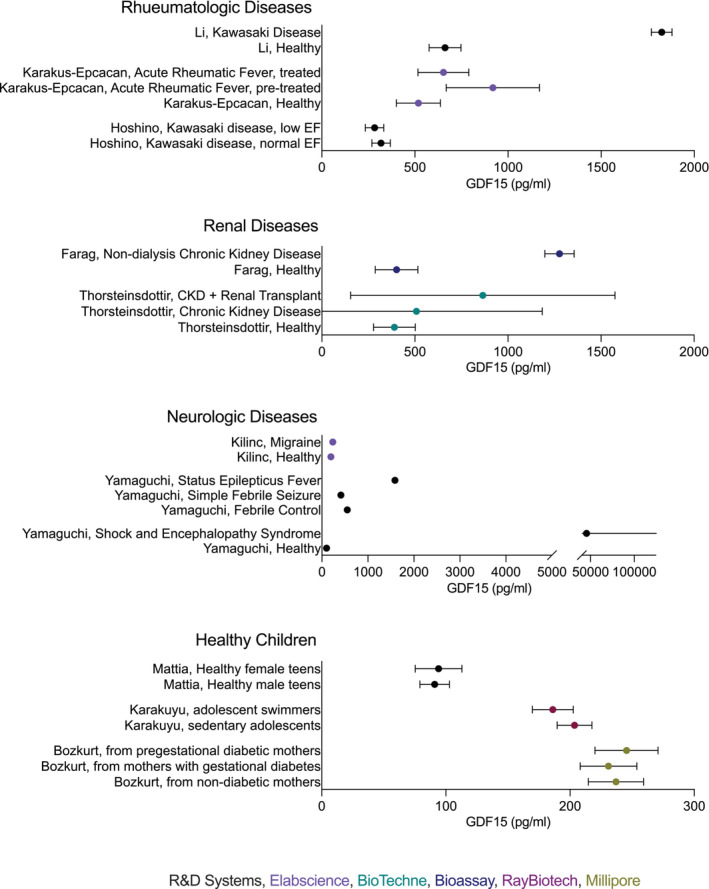
Reported GDF15 levels in rheumatologic, renal, and neurologic diseases and in healthy children. Data are reported as mean ± SD. When the references reported median and interquartile range (IQR), mean and SD were calculated from non‐skewed data or approximated as Median ± IQR.

**FIGURE 5:**
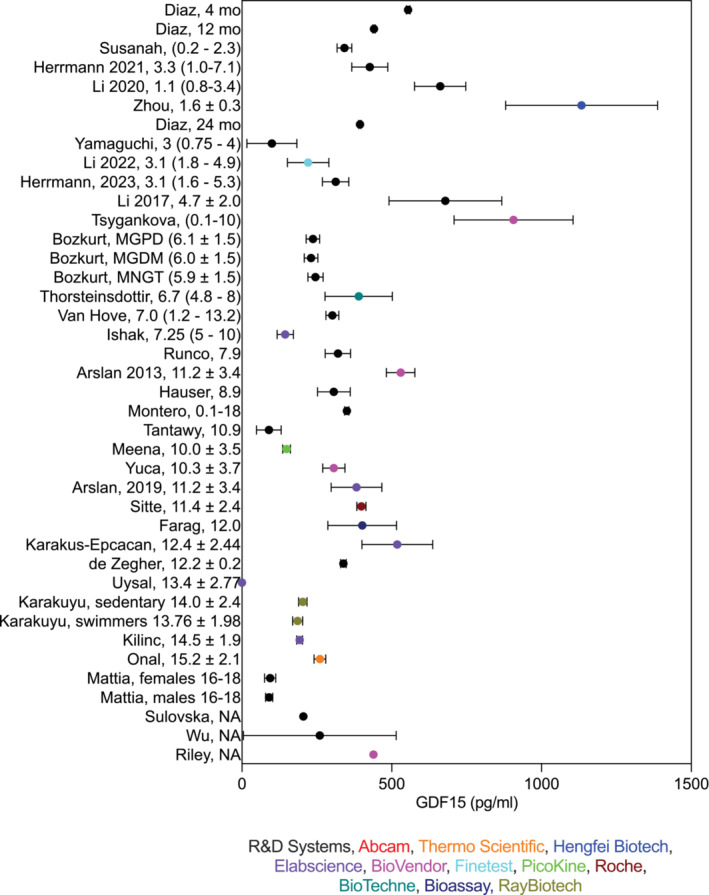
Reported GDF15 levels in healthy children across all studies in Figures 2, 3, 4 organized by age. Data are reported as mean ± SD. When the references reported median and interquartile range (IQR), mean and SD were calculated from non‐skewed data or approximated as Median ± IQR.

For presentation of data in the figures, the five‐number summary method was used to convert mean or median and IQR or range values into mean ± SD (Figures 2, 3, 4, 5) [15, 16, 17, 18]. Skewed (non‐normal) datasets (indicated in Table 1) were also approximated as median ± IQR in the figures per Greco et al. [19]. Original values and statistical data were retained and presented as described in Table 1.

**TABLE 1:** Study population, size, age, GDF15 value and assay used by all included studies (classified by system).

Author	Study population	Sample size(s)	Age	GDF15: healthy control group (H) ^^^	GDF15: study group ^^^	GDF15 assay
Cardiac disorders						
Hauser et al. (2016)	Patients with congestive heart failure (CHF) separated into congenital heart disease (CHD) and cardiomyopathy (CM) subgroups compared to healthy controls	Healthy (H): 89 CHD: 71 CM: 43	H: 8.9 (2.7–14.5) HF: 7.5 (2.4–13.0)	307 (241–501)^$^	CHD: 405 (286–591) CM: 374 (277–707)^$^	R&D Systems
Li et al. (2017)	Patients with systemic‐to‐ pulmonary shunt CHD, grouped by presence or absence of pulmonary arterial hypertension (PAH)	H: 30 CHD alone: 39 CHD + PAH: 46	H: 4.7 ± 2.0 CHD alone: 4.4 ± 2.8 CHD + PAH: 4.8 ± 2.3	611.9 (383–1030)	CHD alone: 890.6 (394.7–1094.3)^+^ CHD + PAH: 1415 (926.7–211.7)* ^,+^	R&D Systems
Zhou et al. (2018)	Infants diagnosed with CHD and CHF compared to healthy infants	H: 80 Mild CHF: 33 Mod CHF: 32 Sev CHF: 35	H: 1.6 ± 0.3 CHF: 1.5 ± 0.3	1134 ± 138.6	All CHF: 1876 ± 167.3* Mild CHF: 1156.9 ± 146.6^+^ Moderate CHF: 1784.7 ± 152.5* ^,+,#^ Severe CHF: 2279.3 ± 168.3* ^,#^	Hengfei Biotech
El Amrousy et al. (2020)	Patients with CHD with or without PAH compared to healthy controls	H: 40 CHD alone: 40 CHD + PAH: 40	H: 1.0 ± 0.4 CHD alone: 1.0 ± 0.4 CHD + PAH: 1.0 ± 0.4	0.15 ± 0.03 nmol/mL	CHD alone: 0.25 ± 0.04 nmol/mL* ^,+^ CHD + PAH: 0.59 ± 0.29 nmol/mL* ^,+^	NA
Kagiyama et al. (2020)	Patients with simple, unrepaired CHD with left to right shunt who underwent cardiac catheterization	69	5.9 [0.1–14]	NA	242.1; [13.6–1116.7]^$^	R&D Systems
Herrmann et al. (2021)	Post cardiac arrest (CA) patients compared to both paediatric intensive care unit (PICU) and healthy controls	H: 32 PICU: 9 CA: 27	H: 3.3 (1.0–7.1) PICU: 3.6 (2.5–10.6) CA: 7.8 (1.1–12.7)	396 (332–547)	PICU: 2122 (1137–2638)* ^,+^ CA: 7089 (3805–13 306)* ^,+^	R&D Systems
Önal et al. (2021)	Patients with previously surgically corrected tetralogy of Fallot (TOF)	H: 40 TOF: 40	H: 15.3 ± 2.1 TOF: 15.2 ± 2.9	260.5 ± 61.2	254.9 ± 67.3	Thermo Scientific™
Van den Bosch et al. (2021)	Patients after Fontan procedure and resulting follow up information	133	Baseline: 13.2 (10.4–15.9)	NA	NA	Olink
Paneitz et al. (2022)	Patients with CHD who underwent cardiac surgery with failure to thrive (FTT) or without FTT	No FTT: 157 FTT: 81	Total: 1.2 [0.2–10]	NA	No FTT: 776 (512–1201)^+,$^ FTT: 1587 (933–2548)^+^	Millipore Sigma
Herrmann et al. (2023)	Paediatric patients post CA compared to healthy controls	H: 20 CA: 111	H: 3.1 (1.6–5.3) CA: 2.6 (0.6–10.8)	313 (311–405)^$^	3230 (1537–10 107)* ^,$^	R&D Systems
Mao et al. (2023)	Patients with pneumonia (PNA) complicated by myocardial damage (MD) versus with simple PNA	PNA alone: 91 PNA + MD: 86	PNA alone: 7.0 ± 1.2 PNA + MD: 7.0 ± 1.5	NA	PNA alone: 457.58 ± 127.58^+^ PNA + MD: 730.78 ± 142.29^+^	Abcam
van der Ven et al. (2023)	Children undergoing Fontan procedure measuring GDF15 levels before surgery and over time after surgery	60	1.1 (0.6–2.6)	NA	NA	Olink
Endocrine disorders						
Yuca et al. (2017)	Obese patients (BMI > 95th percentile) compared to healthy, normal‐weight controls	H: 20 Obese: 39	H: 10.3 ± 3.7. Obese: 10.8 ± 2.7	307 ± 79	314 ± 81	BioVendor
Arslan et al. (2019)	Patients with subclinical hypothyroidism (SH) compared to healthy controls	H: 41 SH: 31	H: 11.2 ± 3.4 SH: 9.6 ± 4.7	382.6 ± 268.2	473.6 ± 337.9	Elabscience
de Zegher et al. (2021)	Non‐obese adolescent girls with PCOS compared to healthy control girls	H: 20 PCOS: 58	H: 12.2 ± 0.2 PCOS: 15.7 ± 0.2	339 ± 20	302 ± 17	R&D Systems
Uysal et al. (2021)	Patients with Type 1 Diabetes Mellitus (T1DM) compared to healthy controls	H: 40 T1DM: 38	H: 13.40 ± 2.77 T1DM: 14.1 ± 2.6	0.563 ± 0.154	0.811 ± 0.304*	Elabscience
Afify et al. (2022)	Patients with T1DM for > 5 years, classified by the presence or absence of microvascular complications (MC)	No MC: 20 MC: 20	Combined: 14.1 ± 2.4	NA	No MC: 250 (220–290)^+^ MC: 390 (300–400)^+,$^	NA
Carreras‐Badosa et al. (2022)	Children with obesity receiving metformin vs. placebo after 24 months	Placebo: 9 Metformin: 9	Range: 6–13	NA	Placebo: 77.5 Metformin: 80.3	R&D systems
Galuppo et al. (2022)	Otherwise healthy patients with BMI > 85th percentile classified by the presence or absence of non‐alcoholic fatty liver disease (NAFLD)	No NAFLD: 89 NAFLD: 86	No NAFLD: 13.1 ± 3.1 NAFLD: 13.6 ± 3.0	NA	NA	R&D Systems
Díaz et al. (2023)	Girls with precocious pubarche and a history of low birth weight assigned to receive metformin or remain untreated for 4 years	Untreated: 15 Metformin: 15	Untreated: 12.0 ± 0.2 (at end of trial) Metformin: 11.9 ± 0.2 (at end of trial)	NA	Untreated: 340 ± 30^+^ Metformin: 488 ± 59^+^	R&D Systems
Zdanowicz et al. (2023)	Obese children with metabolically associated fatty liver disease (MAFLD) compared to obese children without MAFLD	No MAFLD: 25 MAFLD: 31	No MAFLD: 12 (9–16) MAFLD: 12 (11–13)	NA	No MAFLD: 241.08 (210.56–292.28) MAFLD: 277.12 (229.88–312.52)	Cloud‐Clone
Mitochondrial diseases						
Montero et al. (2016)	Patients with mitochondrial disease and non‐mitochondrial myopathies (NMM) versus healthy controls	H: 33 NMM: 19 Mito: 48	H: [0.1–18] NMM: [0.2–18] Mito: [0.1–17]	350.3 ± 20.7	NMM: 349.1 ± 32.2^+^ Mitochondrial disease: 4046 ± 1492* ^,+^	R&D Systems
Dominguez‐Gonzalez et al. (2020)	Patients with early/childhood onset Thymidine Kinase 2 Deficiency	15	[1.8–16]	NA	10 716 ± 2579	R&D Systems
Gojanovich et al. (2021)	Children with perinatally acquired human immunodeficiency virus	No mito: 23 Mito: 23	No mito: 8.7 (5.6, 11.0)	NA	No mito: 484.7 (414.8, 790.3)^+^ Mito: 1441.1 (436.7, 2514.4)^+^	BioVendor
	(PHIV) with or without symptoms of mitochondrial disease		Mito: 12.3 (6.5, 14.7			
Riley et al. (2021)	Patients with mitochondrial disease compared to healthy controls	H: 30 Mito: 56	H:NA Mito: 9.7 [0.8–17]	439	1336*	BioVendor
Tsygankov a et al. (2019)	Patients with mitochondrial and non‐mitochondrial diseases compared to healthy controls	H: 60 Non‐Mito: 127 Mito: 80	H: [0.1–10] All diseases: [0.0–18]	907 (575–1345)	Non‐Mito: 1490 (452.2–5028)* Mito: 4640 (1896–14 064)*	BioVendor
Li et al. (2022)	Patients with primary mitochondrial disorders (PMDs) and non‐mitochondrial neuromuscular disorders (NMDs) compared to healthy controls	H: 50 NMD: 30 PMD: 51	H: 3.1 [1.8–4.9] NMD: 3.5 [2.9–7.0] PMD: 3.0 [0.9–8]	221.2 (117.1–360.6)	NMD: 294.9 (139.0–448.0) PMD: 919.5 (539.5–1670.3)*	Finetest
Van Hove et al. (2024)	Children with mitochondrial or non‐mitochondrial hepatopathies compared to controls healthy controls	H: 186 Biliary atresia:18 α1‐antitrypsin deficiency: 38 Alagille syndrome: 38 Nonalcoholic steatohepatitis: 20 Mito: 36	H: 7.0 (1.2–13.2) Biliary atresia: 8.4 (4.1–15.2) α1‐antitrypsin deficiency: 3.2 (1.0–8.3) Alagille syndrome: 3.2 (1.0–8.3) Nonalcoholic steatohepatitis: 13.2 (10.7–14.5) Mito: 7.0 (0.8–9.3)	Healthy controls: 302 (248–399)	Biliary atresia: 460 (246–798)^+^ α1‐antitrypsin deficiency: 347 (254–525)^&^ Alagille syndrome: 1136 (556–1663)* ^,‐^ Nonalcoholic steatohepatitis: 319 (243–450)^%^ Mito: 2666 (347–8788)* ^+&‐%^	R&D Systems
Hematologic conditions						
Wu et al. (2013)	Patients with hemophagocytic lymphohistiocytosis (HLH) compared to healthy controls	H: 20 HLH: 28	H:NA HLH: 3.7 [0.8–15]	260 [104–649]	1700 [190–2400]*	R&D Systems
Tantawy et al. (2014)	Patients with sickle cell disease (SCD), both Haemoglobin (Hb)	H: 35 HbSS: 22	H: 10.9 [3.2–18]	90 (30–150)	All SCD: 2750 (2150–3350)* HbSS: 2700; [465–4200]*	R&D Systems
	SS and Sβ°, compared with healthy controls	HbSβ°: 13	All SCD: 12.1 [3.2–18]		HbSβ°: 2800 [600–4500] *	
Tantawy et al. (2015)	Patients with β thalassemia intermedia (TI) compared with healthy controls	H: 35 β TI: 35	H: 9.1 [3–17] β TI: 10.2 [3.5–18]	110 (80–200)	1500 (1000–2850)*	R&D Systems
Sulovska et al. (2016)	Patients who have erythrocyte membrane defects (EMD) or are thalassemia carriers (TC) compared to healthy controls	H: 47 EMD: 20 TC: 13	H:NA EMD: [2–18] TC: [3–17]	205 ± 27	EMD: 387 ± 131 TC: 653 ± 545*	R&D Systems
Susanah et al. (2021)	Patients with newly diagnosed β thalassemia major (TM) compared to healthy controls	H: 50 β TM: 41	H: [0.2–2.3] β TM: [0.2–2.0]	342.4 [350.1–437.0]	2095.3 [149.3–9586.3]*	R&D Systems
Meena et al. (2023)	Patients with TM with > 20 lifetime blood transfusions compared to healthy controls	H: 33 TM: 39	H: 10.0 ± 3.5 TM: 10.5 ± 3.7	149.0 ± 36.1	638.7 ± 307.0*	PicoKine
Neonatal						
Olsson et al. (2018)	Otherwise healthy preterm infants (< 28 weeks) observed for the first seven days of life for persistence of a hemodynamically significant PDA	Insignificant: 33 PDA: 14	Insignificant gestational age: 25 + 6 (24 + 4–26 + 5) PDA gestational age: 23 + 4 (22 + 5 to 24 + 1)	NA	Hemodynamically insignificant or resolved PDA: 3361 (2617–4508) *Arbitrary Units* Persistent PDA: 5269 (4010–6091)* *Arbitrary Units*	Olink
Díaz et al. (2020)	Healthy infants who were small‐for‐gestational‐age (SGA) compared to appropriate‐for‐gestational‐age (AGA)	AGA: 70 SGA: 33	AGA gestational age: 39.7 ± 0.1 SGA gestational age: 38.8 ± 0.3	AGA (age at sample): 4 months: 555 ± 25^#^ 12 months: 441 ± 16 24 months: 394 ± 15	SGA (age at sample): 4 months: 469 ± 34^#^ 12 months: 426 ± 25 24 months: 357 ± 15	R&D Systems
Kinoshita et al. (2021)	Newborn infants at gestational age > 33 weeks with birth weight > 1500 g who were hospitalized at a neonatal intensive care unit (NICU)	18	Gestational age: 37.2 (36.9–38.6)	NA	Birth (0 h): 4025.0 ± 1821.8^+‐&^ 2 h: 4061.7 ± 2081.9 6 h: 4402.0 ± 2065.3 2 days: 2922.3 ± 1336.4^+^ 3 days: 1971.3 ± 715.8^‐^ 7 days: 1394.4 ± 507.6^&^	R&D Systems
Zhong et al. (2021)	Extremely preterm infants throughout the first 40 days of life	14	Gestational age: [22 + 6–27 + 4]	NA	NA	Olink
Almudares et al. (2023)	Otherwise healthy preterm infants (< 37 weeks) admitted to the NICU within the first week of life	Gestational age 31–37 weeks: 35 Gestational age < 30 weeks: 17	Average overall gestational age: 30 ± 3.2 weeks	NA	Gestational age 31 and 37 weeks: 3717.5 ± 2336.4^+^ Gestational age < 30 weeks: 7201.1 ± 3880.4^+^	R&D Systems
Oncologic conditions						
Arslan et al. (2013)	Paediatric cancer patients 6 months after completion of anthracycline chemotherapy compared to healthy controls	H: 32 Cancer: 38	H: 8 [3–17] Cancer: 7.46 [1–16]	530 ± 132.14	631 ± 230.2*	BioVendor
Kaya et al. (2016)	Patients with newly diagnosed lymphoma or solid tumour whose protocol includes anthracycline measured over time	20	14 [3–18]	NA	NA	BioVendor
Sitte et al. (2022)	Former paediatric cancer patients ≥ 8 years after diagnosis, compared to healthy controls	H: 50 Cancer: 50	H: 11.4 ± 2.4 Cancer: 16.2 ± 4	399.0 (399.0–453.7)	535.6 (431.9–645.2)*	Roche
Runco et al. (2023)	Children with newly diagnosed cancer versus healthy controls	H: 27 Cancer: 30	H: 7.9 (5.8, 15.9) Cancer: 10.7 (5.1, 16.1)	320.5 (276.6–384.1)	614.6 (420.4–774.2)*	R&D Systems
Infectious diseases						
de Mast et al. (2010)	Patients with asymptomatic malaria or symptomatic malaria compared to healthy children	H: 17 Asymptomatic: 91 Symptomatic: 7	H: 10.9 [8–15] Asymptomatic: 10.9 [6–15] Symptomatic: NA	NA	Symptomatic: Mean: 2467 [940–4500]*	R&D Systems
Manyelo et al. (2021)	Patients with tuberculous meningitis (TBM) with or without stroke compared to	Not TBM: 24 TBM alone: 9	Not TBM: 2.5 (0.8–8.0)	NA	Not TBM: 400 {300–600} TBM‐no stroke: 100 {100–300}^+^ TBM‐stroke: 400 {200–600}^+^	Merck Millipore
	Children with meningitis symptoms that were TB negative	TBM + stroke: 14	TBM alone: 1.25 (0.4–2.3) TBM + stroke: 2.0 (0.9–3.3)			
Majonga et al. (2022)	Patients with PHIV compared to healthy controls	H: 211 PHIV: 195	H: 10.8 ± 2.8 PHIV: 10.7 ± 2.6	NA	NA	Luminex
Ishak et al. (2023)	Children with COVID‐19 compared to healthy controls	H: 72 COVID: 72	H: 7.25 (5–10) COVID: 6.0 (2.38–10.63)	144.66 (89.53–205.8)	614.23 (338.67–1109.33)*	Elabscience
Rheumatologic disorders						
Hoshino et al. (2019)	Patients with a history of Kawasaki Disease (KD) complicated by low ejection fraction (LEF) after symptom resolution and EF normalization compared to patients with previous KD but normal EF	KD alone: 16 KD + LEF: 16	KD alone: 3.6 (2.4–4.5) KD + LEF: 3.7 (2.3–5.4)	NA	KD alone: 318.1 (267.3–359.8) KD + LEF: 283.5 (259.0–351.5)	R&D Systems
Karakuş‐ Epçaçan et al. (2020)	Patients with acute rheumatic fever (ARF) compared to healthy children	H: 25 ARF: 25	H: 12.04 ± 2.44 ARF: 10.98 ± 2.71	518.8 ± 286.2	Pre‐treatment ARF: 918.4 ± 605.7* ^+^ Post‐treatment ARF: 653.1 ± 330.9^+^	Elabscience
Li et al. (2020)	Patients with KD compared to healthy controls	H: 30 KD: 131	H: 1.1 (0.8–3.4) KD: 1.9 (0.9–3.3)	662.1 ± 229.1	1825 ± 322.3*	R&D Systems
Duvvuri et al. (2023)	Patients with Juvenile Dermatomyositis (JDM) compared to healthy controls	H: 22 JDM: 78	H: 13.2 (4.1–19.0) JDM: 9.8	NA	NA	R&D Systems
Renal disease						
Bargenda et al. (2016)	Patients with Chronic Kidney Disease (CKD) on automated peritoneal dialysis or haemodialysis (APD or HD) compared with primary	PNE: 30 APD: 22 HD: 19	PNE:10 (5.5–15.5) APD: 10.0 (4.0–15.5)	NA	NA	R&D Systems
	nocturnal enuresis (PNE) patients		HD: 13.5 (10.5 – 17.0)			
Thorsteinsd ottir et al. (2020)	Patients with a functioning graft at least one year after renal transplantation (Rtx) and patients with CKD compared to a healthy control group	H: 40 CKD: 83 Rtx: 53	H: 6.7 [4.8–8] Rtx: 12.2 [2.3– 18.0] CKD: 10.1 [2.0–17.5]	390 [306–657]	CKD: 508 [183–3279]* ^+^ Rtx: 865 [463–3039]* ^+^	Bio‐Techne
Farag et al. (2023)	Non‐dialysis CKD patients compared to healthy controls	H: 28 CKD: 60	H: 12.0 (10.3–13.0) CKD: 10.0 (8.0–13.0)	401.6 (298.6–594.5)	1277.2 (1072.3–1376.6)*	Bioassay Technology Laboratory
Neurologic disorders						
Yamaguchi et al. (2021)	Patients in the first 24 h of haemorrhagic shock and encephalopathy syndrome (HSES) compared to healthy male controls	H: 5 HSES: 6	H: 3 [0.75–4] HSES: 2.5 [0.25–9]	100.2 (63.1, 130.3)	45 352 (33 547, 112 465)*	R&D Systems
Yamaguchi et al. (2022)	Patients with status‐epilepticus‐ associated‐with‐fever (SEF) compared with control simple febrile seizure patients (SFS) and febrile control patients (FC)	FC: 8 SFS: 8 SEF: 21	FC: 5.0 (3.3–5.5) SFS: 2.0 (2.0–3.0) SEF: 1.6 (1.1–2.0)	NA	FC: 551 (518–670)^+^ SFS: 411 (321–633)^&^ SEF: 1587 (693–2470)^+,&^	R&D Systems
Kilinc et al. (2023)	Female patients with migraines compared to healthy female controls	H: 20 Migraine: 68	H: 14.5 ± 1.9 Migraine: 14.2 ± .8	192.8 ± 20.7	233.7 ± 24.7*	Elabscience
Healthy children						
Bozkurt et al. (2016)	Healthy patients born to mothers with gestational (MGDM) or pregestational (MGPD) diabetic women compared to non‐diabetic mother controls (MNGT) years later	MNGT: 18 MGDM: 32 MGPD: 26	MNGT: 5.9 ± 1.5 MGDM: 6.0 ± 1.5 MGPD: 6.1 ± 1.5	MNGT: 237.0 ± 44.7 MGDM: 231.1 ± 63.2 MGPD: 245.5 ± 62.8	NA	R&D Systems
Karakuyu et al. (2017)	Adolescent male swimmers compared to sedentary adolescents	Sedentary: 29 Swimmers: 26	Sedentary: 13.76 ± 1.98 Swimmers: 14.0 ± 2.4	Sedentary: 203.6 ± 36.8 Swimmers: 186.1 ± 40.7	NA	RayBiotech
Prunicki et al. (2020)	Healthy patients from Fresno, California	100	16.1 ± 2.5	NA	NA	Millipore
Carreras‐Badosa et al. (2021)	Healthy patients' GDF15 levels correlated with IgA and IgG levels	204	8.5 ± 1.8	NA	NA	R&D Systems
Mattia et al. (2023)	Healthy male and female teenagers	Male: 30 Female: 30	[16 – 18]	Male: 91 ± 31.8 Female: 94.1 ± 50.3	NA	R&D Systems

*Note:* ^+, ‐, &, or %^ *p* < 0.05, significant to respectively indicated value.

^a^ Age given in years for the study group(s).

^b^ All values presented as mean ± SD, mean {95% confidence interval}, median (IQR), or median [range] unless otherwise noted.

^ pg/mL.

* *p* < 0.05, significant compared to healthy controls.

$ Skewed data (based on the 5 number summary^15^).

## 3 Results

The systematic review resulted in 1530 studies originally identified with 481 duplicates removed. One thousand and forty‐nine titles and abstracts were screened resulting in exclusion of 931 studies. Of the screened studies, 118 were fully reviewed resulting in 62 included studies (Figure 1). The majority of identified studies were classified as cardiac (*N* = 12), followed by endocrine (*N* = 9), mitochondrial (*N* = 7), hematologic (*N* = 6), neonatal (*N* = 5), oncologic (*N* = 4), infectious (*N* = 4), rheumatologic (N = 4), renal (*N* = 3) and neurologic (N = 3). Five studies of healthy cases were identified and included. Summary data extracted from these publications, including cohort size, age, blood GDF15 levels and assay methodologies, are detailed in Table 1. The R&D Systems Quantikine ELISA was used in most studies (*N* = 31), followed by BioVendor (*N* = 6), Elabscience (*N* = 5), Olink (N = 4) and Millipore (N = 3). Abcam, ThermoScientific, Hengfei Biotech, Cloud‐Clone, Finetest, PicoKine, Luminex, RayBiotech, Roche, Bio‐Techne and Bioassay Tech assays were reported each in one publication and two studies did not report bioassay details. GDF15 concentrations were reported, necessarily omitting relative quantitation metrics derived from Olink and Luminex (Figures 2, 3, 4).

### 3.1 Cardiac Disorders

Two studies compared children with both congenital heart disease (CHD) and heart failure (HF) to healthy children and found higher GDF15 levels in severe HF compared to mild HF and healthy controls [20, 21]. In a population of patients with right‐to‐left (R‐L) shunt CHD, GDF15 was higher in infra‐tricuspid compared to supra‐tricuspid shunts, without correlation to shunt magnitude [22]. Three CHD studies found positive correlation between GDF15 levels and degree of HF [20, 22]. Multiple studies showed contradictory changes in GDF15 after CHD corrective surgery; while one study found no difference between surgically corrected TOF and controls, another found higher GDF15 in patients with surgically corrected CHD with subsequent failure to thrive [23, 24]. In patients undergoing Fontan procedure, GDF15 immediately increased, but returned to normal by one year [25]. Another study showed that lower GDF15 was associated with improved severe event‐free survival after Fontan procedure [26]. In pulmonary arterial hypertension (PAH), oxygen delivery negatively correlates with GDF15 levels in patients with R‐L shunts [22]. Two studies showed PAH‐CHD had higher GDF15 levels compared to both CHD alone and healthy controls [27, 28]. Additionally, increased GDF15 levels were associated with higher morbidity and mortality among patients with PAH‐CHD [27].

GDF15 also appears related to cardiac function in children with other systemic conditions including diabetes and cardiac arrest. Uysal et al. showed GDF15 correlated with diastolic dysfunction in patients with diabetes mellitus [29]. Higher GDF15 levels were found in post‐cardiac arrest patients compared to healthy and paediatric intensive care controls, and higher GDF15 levels correlated with increased mortality and worse neurologic outcomes [30, 31]. Further, among in‐hospital mortalities, cardiac causes of death had higher GDF15 levels than neurologic causes [31]. In a study of patients with thalassemia intermedia, GDF15 levels correlated with cardiovascular disease; and in patients with pneumonia, GDF15 effectively predicted the degree of myocardial damage [32, 33].

### 3.2 Endocrine Disorders

Reports on GDF15 and metabolism demonstrate inconsistent findings. During oral administration of glucose, plasma GDF15 declines over the first 60 min followed by return to baseline [34]. In contrast, two studies examined, and found no clear relationship, between obesity and GDF15 [35, 36]. The lack of correlation between GDF15 and weight change was shown in another study during chemotherapy [37]. However, other examinations of chronic disease do demonstrate a relationship between GDF15 and anthropometric measures or body composition. In a study of patients with CHD, GDF15 was negatively associated with both weight‐ and height‐for‐age [23]. In adolescents with obesity with or without fatty liver disease (FLD), one study showed higher GDF15 levels in concomitant FLD, but another found no difference between these groups [34, 38]. Treatment with metformin increased GDF15 in two studies: a group of obese children and girls with very low birthweight [39, 40].

In addition to obesity, other endocrinopathies reportedly impact GDF15 levels. Two studies describe higher GDF15 in patients with Type 1 Diabetes Mellitus (T1DM) compared to controls [29, 41]. Among patients with diabetes, GDF15 levels were further elevated in those with presence of microvascular complications or HbA1c > 7% [41]. In non‐obese adolescents with polycystic ovarian syndrome (PCOS), De Zegher et al. found no significant difference in GDF15 levels compared to adolescents with normal menses [42]. Upon receiving spironolactone‐pioglitazone‐metformin or oral contraceptive treatment, individuals with PCOS showed higher GDF15 levels with subsequent return to baseline after 6 months off treatment [42]. Finally, no difference in GDF15 level was found in patients with subclinical hypothyroidism compared to controls [43].

### 3.3 Mitochondrial Diseases

Our search yielded five studies comparing children with mitochondrial disease, all showing significantly increased GDF15 levels compared to healthy controls [44, 45, 46, 47, 48]. Similarly, in a group of children with perinatally acquired human immunodeficiency virus (HIV) infections, those with mitochondrial disease had significantly higher GDF15 [49]. However, GDF15 did not correlate with disease severity within mitochondrial disease groups [46, 47, 48]. Compared to a wide variety of other inherited disorders, patients with mitochondrial disease were found to have higher GDF15 levels, highest in mitochondrial hepatopathy [44, 45, 46, 47, 48]. There may be a prognostic utility to combining GDF15 and FGF21 in hepatic mitochondrial disease [45]. When examining cardiac and hepatic diseases in children with and without mitochondrial diseases, no differences were seen in GDF15 levels based on the presence of mitochondrial disease or disease severity [44]. Early or childhood onset of Thymidine Kinase 2 Deficiency, a mitochondrial disease, demonstrated 30‐fold higher GDF15 levels compared to controls, with improvement in clinical outcomes and decreases in GDF15 after treatment with oral deoxynucleosides [50]. Three of these studies utilized non‐mitochondrial myopathy, such as Duchene Muscular Dystrophy or Spinal Muscular Atrophy, patients as control groups and found similar to lowered GDF15 levels compared to healthy controls [46, 47, 48].

### 3.4 Hematologic Conditions

Four studies evaluated GDF15 levels in patients with alpha‐ or beta‐thalassemia and all found elevated GDF15 compared to controls [33, 51, 52, 53]. Newly diagnosed beta‐thalassemia (βT) major showed GDF15 values up to six times higher than healthy controls, while βT intermedia and βT minor also demonstrated increased GDF15 [33, 51, 52]. Further, individuals with βT major with over 20 lifetime blood transfusions, had four times higher GDF15 than healthy controls [53]. In children with sickle cell disease, both Haemoglobin (Hb) SS and HbSβ° have significantly elevated GDF15 levels [54]. In this study, GDF15 level was associated with elevated serum ferritin (> 2500 μg/L), previous stroke and splenectomy, but not with frequency of sickle cell crises, PAH, or hydroxyurea usage in these patients [54]. Hemophagocytic lymphohistiocytosis (HLH), also results in elevated GDF15 levels, over six times greater than controls [55]. In these hematologic processes, GDF15 positively correlates to transfusions, ferritin levels, transferrin saturation and lactate dehydrogenase [33, 52, 53, 54, 55].

### 3.5 Neonatal

GDF15 levels are known to increase in the pregnant woman throughout pregnancy, as well as significant production by the placenta itself. While there are reports of placental transfer of GDF15 from mother to foetus, conflicting views exist on the impact of GDF15 from maternal, placental and foetal sources. Several perinatal and neonatal studies show gestational age at birth and birth weight negatively correlate with GDF15 levels [56, 57]. Almudares et al. described an average of 475.0 pg/mL decrease in GDF15 at birth for each additional gestational week [56]. GDF15 levels remain relatively stable throughout the first 6 h of life, then drop significantly from the second day of life through the first several days to weeks, with one study demonstrating an average decline of 118.7 pg/mL per day [56, 57, 58]. In the first four months of life, infants with lower birth weights for gestational age have more rapid drops in GDF15 [59]. Increased GDF15 levels are also associated with pathologic structural cardiac changes and adverse respiratory outcomes in neonates [56, 60].

### 3.6 Oncologic Conditions

GDF15 levels in paediatric oncology have been studied primarily in the setting of anthracycline‐induced cardiotoxicity. Two studies showed elevated GDF15 levels in paediatric patients with cancer who had received anthracycline chemotherapy treatment months to years earlier [61, 62]. Further, in newly diagnosed oncologic patients receiving anthracyclines, GDF15 levels increased, but returned to the patient's baseline six months after the final dose of anthracycline [9]. Runco et al. measured GDF15 levels at diagnosis (prior to treatment) and reported elevated GDF15 levels in paediatric patients with cancer, which persisted through three months of treatment. No correlation was found between GDF15 and anthropometric changes at three months but few patients on the study had significant changes in anthropometric measures [37].

### 3.7 Infectious Diseases

Identified studies examined GDF15 in malaria, and tuberculosis, as well as the studies involving children with HIV described in the mitochondrial section above. Majonga et al. found children with perinatally acquired HIV had a significantly increased level of GDF15, which was associated with worse LV diastolic dysfunction and hypertrophy [63]. A study by de Mast et al. found patients with asymptomatic *Plasmodium falciparum* and *Plasmodium vivax* malaria had similar GDF15 levels as healthy children. However, children with symptomatic malaria had increased GDF15 levels [64]. Tuberculous meningitis was also shown to correlate with increased GDF15 levels, which were further elevated in those with radiographic evidence of a stroke [65]. Paediatric patients with COVID also showed elevated GDF15 levels, further elevated with severe disease or cardiac symptoms [66].

### 3.8 Rheumatologic Conditions

All three rheumatologic studies during active disease showed increased GDF15 [67, 68, 69]. Children with both newly diagnosed Kawasaki Disease (KD) and rheumatic fever had elevated GDF15 levels, but returned to normal after treatment, even in the setting of carditis [67, 68, 70]. In KD, patients with concomitant coronary artery lesions (CAL) had significantly higher GDF15 levels than those without. Further, GDF15 values > 2003 pg/mL, predicted CAL development at high sensitivity and specificity [67]. Similarly, GDF15 was elevated in Juvenile Dermatomyositis and correlated with disease severity [69]. GDF15 levels were positively associated with inflammatory markers such as erythrocyte sedimentation rate (ESR) and C‐reactive protein (CRP) in multiple studies [67, 70].

### 3.9 Renal Disease

GDF15 has also been of interest in renal transplantation. Levels are significantly higher in children post‐renal transplantation with functioning renal grafts, compared to those with chronic kidney disease (CKD), though both have levels higher than controls [71]. Further, plasma GDF15 is negatively associated with renal function in both transplant and CKD populations [71]. Higher GDF15 values were correlated to anaemia among patients with CKD, with values greater than 723 pg/mL suggestive of anaemia [72]. In Bargenda et al., children with CKD on automated peritoneal dialysis (APD) or haemodialysis (HD) were found to have increased GDF15 levels. Additionally, pre‐dialysis GDF15 levels were higher in patients with HD compared to patients with APD, and these levels further elevated after a HD session [73].

### 3.10 Neurologic Disorders

In the three identified studies of children with neurologic conditions, GDF15 levels were significantly elevated. Children with haemorrhagic shock and encephalopathy syndrome within the first 24 h of presentation were found to have GDF15 levels elevated over four hundred times controls [74]. Status‐epilepticus‐associated‐with‐fever also yields significantly elevated GDF15, which was a positive predictor of further sequalae [75]. This trend continued in paediatric migraines as patients were found to have elevated GDF15 compared to controls [76].

### 3.11 Healthy Children and Controls

Five studies evaluated GDF15 levels in healthy children. Mattia et al. found no difference in GDF15 between male and female teenagers (mean 92.55 pg/mL) [77]. Similarly, GDF15 levels were similar between sedentary‐ and swimmer‐adolescents (mean 195.33 pg/mL) [78]. A retrospective study of healthy children found no significant differences of GDF15 based on the presence or absence of maternal diabetes [79]. Another study found GDF15 levels were one of the top predictors of recent exposure to air pollution [80]. Finally, IgG and IgA were shown to correlate with GDF15 levels, a trend which became stronger for individuals with a larger body mass index (BMI) [36]. Among studies of healthy children and healthy control groups, after removing the errata of Uysal et al., (a total of 39 studies) average GDF15 values ranged from 90 to 1134 pg/mL, with mean 343.8 pg/mL (SD 221.0 pg/mL) (Table 1; Figures 2, 3, 4). This can be compared to established adult medians of 537 pg/mL and 628 pg/mL in adults under the age of 30 and 2152 pg/mL and 1847 pg/mL in adults over the age of 80 (male and female, respectively) [81].

## 4 Discussion

**FIGURE 6:**
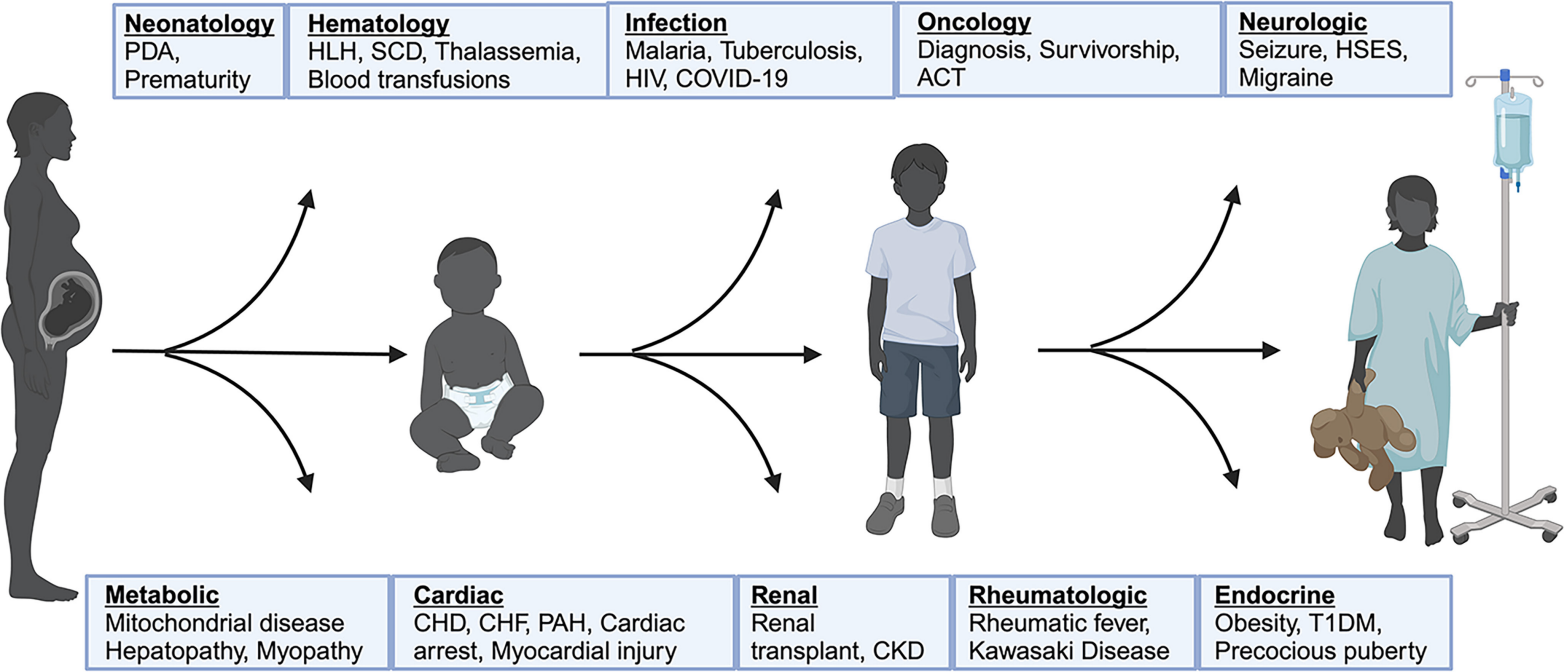
Paediatric conditions demonstrating elevated GDF15. Abbreviations: HIV = human immunodeficiency virus; PDA = patent ductus arteriosus; T1DM = Type 1 Diabetes mellitus; CHD = congenital heart disease; CHF = congestive heart failure; PAH = pulmonary arterial hypertension; HLH = Hemophagocytic lymphohistiocytosis; SCD = sickle cell disease; CKD = chronic kidney disease, ACT = anthracycline cardiac toxicity; HSES = hemorrhagic shock and encephalopathy syndrome. Figure was created with BioRender.com.

GDF15 represents a potential diagnostic and therapeutic target for a variety of conditions in children and, to our knowledge, this is the first comprehensive paediatric review of studies examining circulating GDF15. As of February 2024, we identified 62 studies of primary literature measuring circulating levels of GDF15 in paediatric patients, all published within 20 years. Numbers of paediatric studies pale in comparison to adult publications examining GDF15. Further, unlike adults, children lack established, widely accepted GDF15 reference ranges [81]. While a majority of paediatric studies explored GDF15 in cardiac, endocrine and mitochondrial pathologies, its multifactorial role is evident through its diverse effects upon multiple body systems (Figure 6).

The identified studies suggest that GDF15 is elevated in pathologic heart conditions, consistent with adult studies and preclinical models [7, 82]. Rapid elevation after cardiac procedures indicates a rapid rise of GDF15 to cardiac stressors, and subsequent return to baseline demonstrates its acute, yet temporary nature of hypersecretion [25]. However, separately from intrinsic cardiac disease, GDF15 may be a predictive myocardial biomarker for cardiac injury or flares chronic, systemic inflammatory conditions. Studies suggest acute and chronic GDF15 may result in systemic damage in addition to cardiotoxicity [9, 62, 67, 68, 70]. In resolving immunologic conditions, GDF15 levels return to baseline along with heart function, but in various pathologies such as post‐cardiac arrest and CHD, GDF15 levels above a certain threshold appear to be associated with irreversible, detrimental cardiovascular effects. Compared to other novel cardiac inflammatory biomarkers such as glycoprotein acetyls, both have prognostic and diagnostic properties in cardiac disease, but differ in their specificity of inflammatory markers towards other bodily systems [83]. Through comparison of these levels in various inflammatory states, more direct relationships and even causality may be able to be determined to elucidate their specific and more general effects on the body.

GDF15 is a strong prognosticator of all‐cause mortality in adults with heart disease or on haemodialysis, but additional studies are needed to better characterize GDF15's role as a biomarker or mediator of paediatric cardiac and renal disease [84]. Unfortunately, there are no paediatric studies assessing changes in GDF15 over time as predictors of morbidity and mortality as have been established in the adult population. It has been demonstrated in the literature that GDF15 levels are not only elevated in paediatric patients with CKD, but further increase after haemodialysis sessions, potentially offering insight into the independent effect of GDF15 from kidney function alone [6, 73]. Understanding the destructive nature of supraphysiologic GDF15 levels without discounting any potential benefit in the acute response is important to better characterize its role and identify interventions.

GDF15's role in metabolism may prove critical to better understand diabetes and childhood obesity. The immediate drop in GDF15 after glucose administration indicates a possible relationship with glucose metabolism. Additionally, modulators of glucose uptake, can change GDF15 levels, as was shown with the increase in GDF15 seen with metformin therapy [39, 40]. Adult and mouse studies of starvation and obesity show elevated GDF15 at both very high and very low weights, hypothesized secondary to inflammatory changes and stress signalling [7]. This potential role at both extremes of weight highlights the importance of understanding and studying GDF15 in a disease‐specific context. For children, GDF15 elevations appear to be associated with microvascular and intrahepatic changes related to obesity, but there was not a direct relationship observed between paediatric obesity and GDF15. There were no studies that we found which examined GDF15 in paediatric patients with extremely low BMIs, highlighting a significant gap in paediatric literature [7]. It remains to be seen if GDF15's association with T1DM is causal or related to the secondary systemic inflammation seen with diabetes, but better understanding of the role of GDF15 in glucose metabolism and diabetes is important and warrants further investigation.

Previous adult and in vitro studies show GDF15's role as a mitokine, a soluble factor generated in response to mitochondrial stress and as such may serve in diagnosing, prognosticating and/or treating mitochondrial disease [48, 49]. As evidenced by this review, there is great physiologic heterogeneity in mitochondrial disorders with varying severity in muscle, liver and heart disease severity. It is clear that paediatric mitochondrial diseases demonstrate GDF15 elevation, greatest in patients with hepatic disease. Whether GDF15 drives further systemic damage in mitochondrial disorders or is simply a byproduct of systemic damage remains to be seen. Further, in the setting of neurologic injury, elevated GDF15 acutely confers a neuroprotective state, emphasizing the importance of disease specific study of GDF15 including treatment modifying interventions. Regardless, recent treatment advances specifically in mitochondrial diseases have shown promise in concurrently lowering GDF15 levels and mediating systemic damage, suggesting that GDF15 antagonism may provide benefit in some aspect of systemic mitochondrial disease.

GDF15 has been well described in adult and animal models of disease tolerance, most recently demonstrating its correlation with COVID‐19 severity [85]. As in many systemic diseases, there is a paradoxical benefit and detriment to inflammation, where primary inflammation helps the body fight off infection, but an overzealous immune response becomes detrimental. A prime example is in HLH, where a dysregulated, uncontrolled immune response, prompted often by cancer or infection, causes systemic damage further complicating the disease state. GDF15 further correlates with serum ferritin, a known marker of HLH disease severity [55]. The hyperinflammatory response may disturb the balance of the host defence system and negatively impact disease tolerance, posing deleterious and life‐threatening side effects [85]. Thus, beyond a prognostic usage in inflammation, there also may be a role for GDF15 in disease therapeutics to disrupt the uncontrolled immune response seen in certain systemic diseases.

To emphasize the dual nature of GDF15's protective and detrimental roles, several adult oncologic studies have suggested that GDF15 is protective against early‐stage or development of cancer, but also promotes tumorigenesis and metastasis, in later‐stage disease [6]. Circulating GDF15 and expression of GDF15 in tumour tissue have been shown to increase cancer related cachexia and novel data has shown GFRAL antagonism can reverse cachexia in mice [6]. Unfortunately, study of GDF15 in paediatric oncology is mostly limited to the long‐term cardiotoxicity in childhood cancer survivors who received anthracycline therapy without dedicated disease‐specific study of GDF15's role in development, progression, or treatment resistance. One pilot study showed GDF15 elevation in newly diagnosed patients with cancer compared to healthy controls but fails to account for paediatric tumoral differences and unique molecular profiles compared to adults [37]. Further research must be done in this area, specifically in paediatric cancers with the highest risk of cachexia, to further elicit the effect of tumour type and treatment on GDF15 and subsequent development of cachexia.

Neonatal and perinatal levels of GDF15 have also been identified as an area of interest in metabolic and developmental outcomes. In pregnant women, GDF15 levels increase throughout pregnancy, originally thought to correlate with foetal levels [56]. Yet, the only known study comparing GDF15 levels in preterm infants shows an inverse correlation between GDF15 levels and gestational age [56]. Whether this reflects foetal GDF15 levels at different gestational ages, due to increased physiologic stress of prematurity, or another process remains to be seen. Among a healthy population, neonates have higher GDF15 than children or adults. In part, this is likely due to high levels of placental GDF15 production and transmission to the foetus, but with a short half‐life (approximately 3 h), there appears to be neonatal generation of circulating GDF15 as well [57]. Regardless of birth levels and prematurity, GDF15 decreases over the first few days to months of life, but little is known about trends in healthy children beyond that.

Recent trends of increasing rate and severity of paediatric obesity are concerning for development of further health complications, identifying physical activity and diet as first‐line modifiable risk factors to limit obesity [86]. Much of the GDF15 obesity and exercise data comes from adult or animal literature, describing GDF15 correlations with bouts of exercise, lower BMIs and decreased appetite [4, 6]. As data has shown a U‐shaped curve of inflammation in response to varying intensities of exercise, with both sedentariness and vigorous physical exercise increasing, but light exercise decreasing inflammation, GDF15 levels should be examined on this continuum to establish treatment protocols [87]. Further, adult data has shown the increase in GDF15 associated with an exercise program is related to the reduction in fat mass and increase in insulin sensitivity [88]. A novel, long acting GDF15 receptor agonist was shown to cause decrease in appetite and body weight in overweight mice and adult individuals [11]. While paediatric data is limited on this subject, GDF15 mimetics offer a potential therapeutic target to reduce childhood obesity along with exercise and dietary interventions.

The lack of normalized GDF15 values for healthy children is clearly a barrier to better understanding its role. Studies characterizing normal levels based on age, biologic sex and pubertal status in healthy children can help us better define the role of GDF15 in normal physiology and small factors that account for differences between persons [89]. We stress the need for established reference ranges, stratified by age, in the paediatric population as is widely accepted in adult literature. Currently, BMI, immunoglobulin levels and exposure to air pollutant are known associates of elevated GDF15 levels in children [36, 80]. In healthy adults, the range of GDF15 levels is much greater in females than males, a relationship of unclear aetiology, which has not been similarly demonstrated in children [81]. Many of the factors affecting GDF15 levels in “healthy adults” such as smoking and cholesterol are related to compounded inflammation and immunologic activity over time [81]. Therefore, determining baseline levels in children and trending longitudinal studies can help us better understand the interplay of genetics and external factors on GDF15 as well as its long‐term effects on the body. Then, with a better understanding of the physiologic levels, the pathophysiologic implications of GDF15 in diagnosis, progression and therapeutics of paediatric diseases will become clearer.

## 5 Limitations

This review of the literature should be interpreted in the setting of several limitations. Despite the comprehensive and systematic approach, we created strict inclusion criteria to present the most clinically relevant areas on which future GDF15 study should focus. Doing so may exclude potentially relevant research. Secondly, we describe considerable heterogeneity among the study designs, measurement methodologies and clinical outcomes of interest in a wide array of heterogeneous diseases studied. Thus, the different assays used, and clinical contexts require caution when interpreting the specific values. At least one study has demonstrated common biologically active GDF15 variants may be underestimated by certain assays [14]. Additionally to heterogeneity in the assays and methodologies, some studies demonstrated normal distribution while others featured wide ranges or extreme outliers. We have attempted to summarize the data in a way that gives an impression of GDF15 in paediatric diseases, but recognize the need to examine the nuance of each study before drawing more specific conclusion. As such, this review advocates for larger, prospective studies with disease‐specific methodologies and clinically relevant outcomes to fully understand the potential therapeutic implications of GDF15. Finally, mechanistic understanding of GDF15 in paediatric disease is important. While we have summarized patient populations in which GDF15 appears to mediate disease, exact mechanisms and the differences between tissue expression and circulating GDF15 must be further studied before moving forward with GDF15 antagonists as potential therapeutics.

## 6 Conclusion

GDF15 is an important mediator in many paediatric diseases, but remains to be well defined. This review of the literature provides the first comprehensive description of existing paediatric GDF15 literature and demonstrates the need for more detailed disease‐specific study of GDF15. A key first step would be characterizing normal GDF15 levels in children based on age, biologic sex and pubertal status. Doing so will better define GDF15's role as a diagnostic marker of, mediator of, and potential therapeutic target in childhood diseases.

## Ethics Statement

The manuscript does not contain original clinical studies or patient data. All authors listed on a manuscript comply with the Ethical guidelines for authorship and publishing in the Journal of Cachexia, Sarcopenia and Muscle.

## Conflicts of Interest

Teresa Zimmers is jointly appointed to Oregon Health & Science University and the United States Department of Veterans Affairs and in the past 10 years has served as an advisory board member for Emmyon, Inc. and PeleOS, LLC and has consulted for Pfizer, Inc. Daniel Runco has consulted for Day One Biopharmaceuticals and Pfizer, Inc. during the past 2 years. The other authors have no conflicts of interest to disclose.

## Supporting information


**Appendix S1** Development of the research question in the PICO format.
