# Supplementary material for: Circulating Growth Differentiation Factor 15 (GDF15) in Paediatric Disease: A Systematic Review
Source: J Cachexia Sarcopenia Muscle. 2025 Feb 28;16(2):e13712. doi: 10.1002/jcsm.13712 (PMC11870081; doi:10.1002/jcsm.13712)
Supplement: Supplementary file 1 — Appendix S1 Development of the research question in the PICO format. [file JCSM-16-e13712-s001.pdf]

Supporting Information Appendix S1: Development of the research question in the PICO format.

| | |
|---|---|
| Population | Children (synonyms should include other words that the search includes such as pediatric, childhood, etc.) |
| Intervention | GDF15 (includes synonyms listed in the introduction/methods section for aliases for GDF15) |
| Comparison | No gold standard exists for the diagnostic or therapeutic implications of GDF15 |
| Outcomes | Any diagnostic outcome or disease-based study of GDF15 in pediatrics |
